# A genome-wide resource of cell cycle and cell shape genes of fission yeast

**DOI:** 10.1098/rsob.130053

**Published:** 2013-05

**Authors:** Jacqueline Hayles, Valerie Wood, Linda Jeffery, Kwang-Lae Hoe, Dong-Uk Kim, Han-Oh Park, Silvia Salas-Pino, Christian Heichinger, Paul Nurse

**Affiliations:** 1 Cell Cycle Laboratory, Cancer Research UK, London Research Institute, 44 Lincoln's Inn Fields, London WC2A 3LY, UK; 2 Cambridge Systems Biology Centre and Department of Biochemistry, University of Cambridge, 80 Tennis Court Road, Cambridge CB2 1GA, UK; 3 Department of New Drug Discovery and Development, Chungnam National University, Yusong, Daejeon, South Korea; 4 Aging Research Center, Korea Research Institute of Bioscience and Biotechnology, Yusong, Daejeon, South Korea; 5 Bioneer Corporation, Daedeok, Daejeon, South Korea; 6 Laboratory of Yeast Cell Biology and Genetics, Rockefeller University, 1230 York Avenue, New York, NY 10021-6399, USA; 7 Centro Andaluz de Biología del Desarrollo, CSIC/Junta de Andalucia/Universidad Pablo de Olavide, Carretera de Utrera, km 141013 Sevilla, Spain; 8 Department of Developmental Genetics, Institute of Plant Biology, University of Zürich, Zollikerstrasse 107, 8008 Zürich, Switzerland

**Keywords:** genome-wide gene deletion resource, cell cycle, cell shape, fission yeast

## Abstract

To identify near complete sets of genes required for the cell cycle and cell shape, we have visually screened a genome-wide gene deletion library of 4843 fission yeast deletion mutants (95.7% of total protein encoding genes) for their effects on these processes. A total of 513 genes have been identified as being required for cell cycle progression, 276 of which have not been previously described as cell cycle genes. Deletions of a further 333 genes lead to specific alterations in cell shape and another 524 genes result in generally misshapen cells. Here, we provide the first eukaryotic resource of gene deletions, which describes a near genome-wide set of genes required for the cell cycle and cell shape.

## 2. Introduction

Understanding how cells reproduce and how they generate their shape are two major goals in eukaryotic cell biology. These two processes are related because, during the cell cycle, cells duplicate cellular components and reproduce their cell structure in space to generate two daughter cells. A key function of the cell cycle is to ensure accurate replication and segregation of the genome, because errors in genetic transmission can cause mutations and chromosomal rearrangements that may lead to cell death or disease. Failure to accurately reproduce and maintain cell shape can disrupt tissue architecture or influence cell motility and may also lead to cell death or disease. Given the importance of these two processes for cell biology, we have generated a genome-wide resource, cataloguing the genes that when deleted disrupt the cell cycle or cell shape in the fission yeast *Schizosaccharomyces pombe*. This is the first such resource that qualitatively describes a near complete set of genes required for these processes in a eukaryotic organism.

Fission yeast is very amenable for investigating the cell cycle and cell shape [1–3] and has been used extensively for cell cycle studies for many years [4]. It is a rod-shaped, unicellular eukaryote that grows by apical extension and divides by medial fission and septation. This regular cell shape has made fission yeast a very useful organism to identify genes involved in the cell cycle and the generation and maintenance of cell shape. Mutants can easily be identified by visually screening for cells that divide at a longer or shorter length compared with wild-type (WT) (cell cycle defect), or that do not have a rod-shape (cell shape defect). A long cell phenotype is generated if cells are blocked or delayed in cell cycle progression because they continue to grow but fail to divide and thus become elongated. However, not all genes required for the cell cycle show a long cell phenotype when deleted. For example, genes encoding checkpoint proteins that are not required during a normal cell cycle may have a WT deletion phenotype and genes required for mitosis often arrest with a more irregular, non-elongated cell shape [5]. The long cell phenotype is easily identified by visual screening and is definitive for cells blocked or delayed in cell cycle progression through the G1, S, G2 and cytokinesis phases of the cell cycle [6]. We have therefore focused on identifying all genes with this phenotype when deleted, to determine the majority of genes required for progression through interphase or cytokinesis in fission yeast.

Genes important for the generation of cell shape are also easily identified, because mutants deleted for these genes lose the normal rod-shape. These cells exhibit a range of phenotypes, including rounded or stubby, curved, branched, skittle-shaped or more generally misshapen [2,3]. The penetrance of these mutant phenotypes can be quite variable; in some cases, the majority of cells have the same altered cell shape, whereas in others the phenotype is of lower penetrance with fewer of the cells exhibiting the phenotype.

Fission yeast currently has 5059 annotated protein coding genes, and a genome-wide deletion collection has been constructed with 4836 genes deleted [7]. In this study, we have systematically visually screened the deletion collection to identify genes required during interphase of the cell cycle, cytokinesis and for cell shape. This resource complements and extends earlier studies using gene deletions in budding yeast [8,9] and RNAi-based gene knockdowns in metazoa [10–15]. Although the budding yeast gene deletion collection has been extensively investigated, it has not been subjected to a systematic screen for cell cycle genes such as that carried out here, while metazoan RNAi cell cycle studies have shown only limited reproducibility [12]. Given that 3397 (67.14%) of the fission yeast protein coding genes have identifiable orthologues in metazoa (http://www.pombase.org), the genome-wide resource provided here will help to identify cell cycle and cell shape genes in other eukaryotes, including humans.

## 3. Results

### 3.1. Screening of the haploid deletion mutants

**Figure 1.**
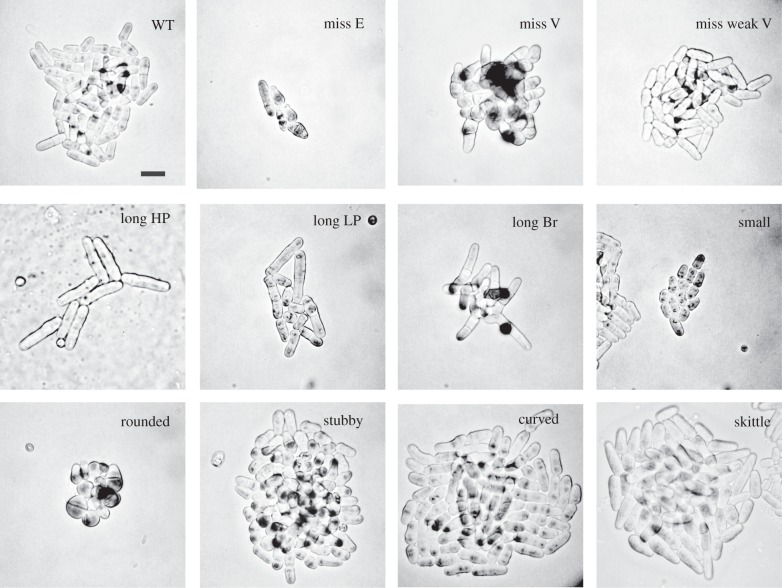
Cell shape categories. Examples of the 11 major cell shape phenotype categories described in this study. For each deletion, mutant cells are shown as observed during the screen. Definitions of each phenotype can be found in the electronic supplementary material 1, table S4. Scale bar (shown in WT) = 10 µm.

**Figure 2.**
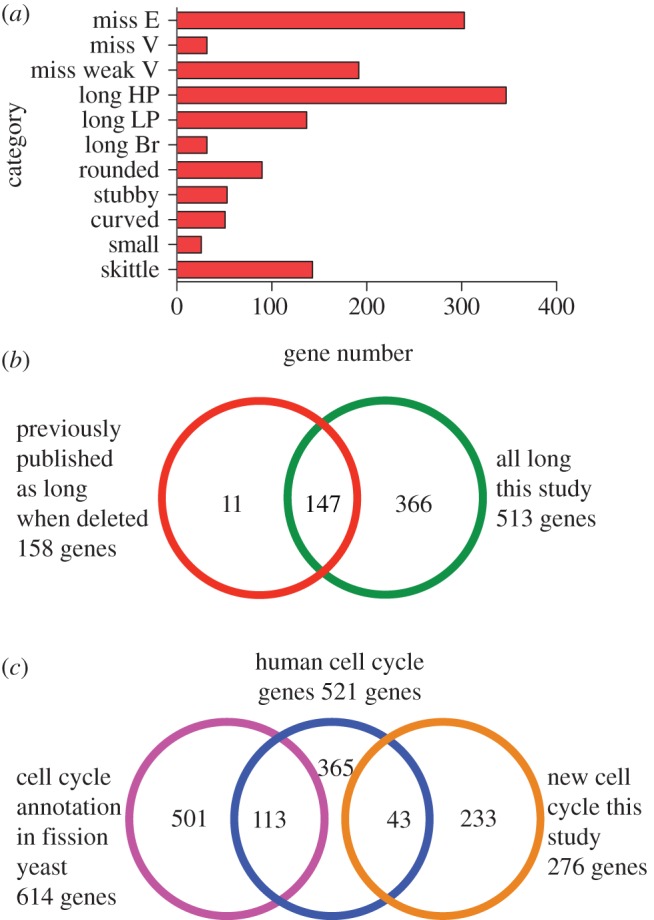
Distribution of cell shape genes. (*a*) Distribution of 1395 genes with a cell shape deletion phenotype among the 11 cell shape categories. (*b*) Overlap of *long* gene set identified in this study (513 genes, green circle) with a set of previously published genes with a long deletion phenotype (158 genes, red circle). For further details see the electronic supplementary material 1, table S8. (*c*) Overlap between (i) 521 fission yeast orthologues of human genes with an RNAi cell cycle phenotype (blue circle), (ii) 614 genes with a mitotic cell cycle annotation in fission yeast (pink circle) and 276 new cell cycle genes from this study (orange circle). For further details see the electronic supplementary material 1, tables S8 and S11.

We have microscopically examined and described the deletion phenotypes of 4843 haploid gene deletion mutants of both essential and non-essential genes, after sporulating diploid heterozygous deletion mutants and germinating haploid spores on rich medium plates (see §5.1 for details of the screen and electronic supplementary material 1, tables S1 (column G)–S3). Mutants were classified to one of 11 cell shape phenotypes together with three additional categories, namely: *WT*; arrested as normal spores (*spores*); or arrested as normal germinated spores (*germination*) (figures 1 and 2*a* and table 1; electronic supplementary material 1, table S1, column H), making 14 phenotype categories in total (see §5.1). These phenotypes were mapped to terms in the Fission Yeast Phenotype Ontology (http://www.obofoundry.org/cgi-bin/detail.cgi?id=fypo) (see the electronic supplementary material 1, table S4), and the gene list for each category can be found in the electronic supplementary material 1, table S5*a–n*. All 4843 deletions were assigned to only one of these 14 categories for analysis. The variation in the phenotype for mutants in a particular category can be found in the phenotype description in the electronic supplementary material 1, table S1, column G. For example, some long mutants may be slightly curved, and T-shaped cells were observed in a subset of curved cells.

**Table 1.** Summary of phenotype categories. Fourteen broad phenotype categories were used for analysis, and the number and dispensability of genes in the different categories is shown. Each gene is classified by a single phenotype category based on the most penetrant or strongest deletion phenotype. Cells were only described as wild-type (WT) when no other phenotype was observed. The classifier for each gene can be found in the electronic supplementary material, table S1, column H and gene dispensability in table S1, column I. All genes are included only once and cell phenotype terms from the electronic supplementary material 1, table S4 that are not included as separate phenotypes are subsets of one of these categories, for example, a T-shaped mutant is included in the *curved* category as curved is the most penetrant phenotype for this type of cell shape mutant.

phenotype categories for analysis	total genes	essential genes	non-essential genes
WT	3041	0	3041
spores	184	184	0
germination	223	223	0
misshapen essential	302	302	0
misshapen viable	31	0	31
misshapen weak viable	191	0	191
long high penetrance	346	215	131
long low penetrance	136	106	30
long branched	31	20	11
rounded	89	55	34
stubby	52	8	44
curved	50	11	39
small	25	14	11
skittle	142	129	13
total genes analysed	4843	1267	3576

Gene Ontology (GO) term enrichment of biological processes and cellular components for these 14 phenotype categories is shown in tables 2–4 (see the electronic supplementary material 1, table S6*a–n* and table S7*a–n* for the complete results). There are 643/4843 genes with no GO process annotation. Of these 643 ‘unknowns’, 574 (89.2%) have a WT deletion phenotype. This means that most genes showing one or more of the 13 other deletion phenotypes are assigned a biological process either by inference from other organisms or because they have been partially characterized in fission yeast. However, their cellular shape is often not part of that characterization.

**Table 2.** GO cellular processes for all phenotype categories. A summary of the GO analysis to identify genes annotated to cellular processes enriched within particular phenotype categories. The enrichment results were mapped to ‘GO slim’ (high level) terms covering most biological processes observed in fission yeast to give a broad view of the ontology content of the genome-wide gene deletion dataset. For details see §5.5.2 and the electronic supplementary material 1, table S6*a–n* and table S14. The total dataset is 4843 genes. Footnotes are denoted by a–n. Red colour denotes enriched *p* ≤ 0.001; orange denotes moderately enriched *p* ≤ 0.01; light orange denotes weakly enriched *p* ≤ 0.1: light blue denotes no enrichment *p* ≤ 1; blank denotes number of genes is 0.

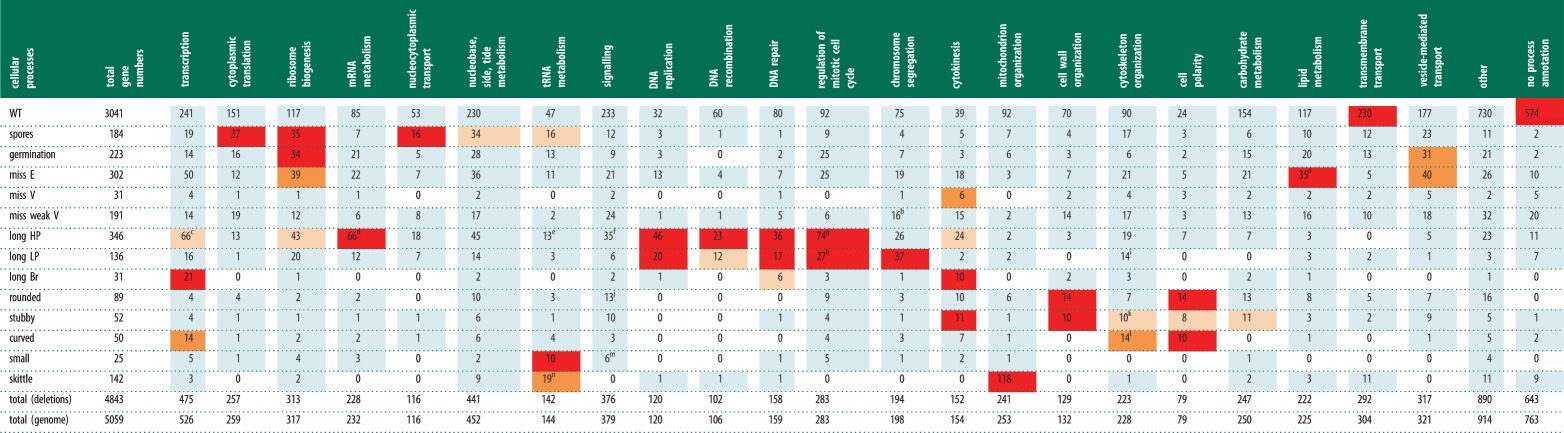

^a^Includes 15/29 genes annotated to glycosylphosphatidylinositol (GPI) anchor biosynthesis, a descendent of lipid metabolism (*p* = 1.75 × 10^−8^).

^b^Includes 10/41 genes annotated to attachment of spindle to microtubules, a descendent of chromosome segregation (*p* = 0.001).

^c^Includes 11/26 genes annotated to histone deacetylation, a descendent of transcription (*p* = 0.00054).

^d^Includes 53/118 genes annotated to nuclear mRNA splicing, via spliceosome, a descendent of mRNA metabolism (*p* = 3.7 × 10^−27^).

^e^Includes 5/6 subunits of the elongator complex involved in tRNA wobble uridine modification.

^f^Includes 11/25 genes annotated to the septation initiation signalling cascade (*p* = 0.00013), and 5/15 genes annotated to the stress activated protein. Kinase signalling cascade, 4/19 genes annotated to TOR signalling and 3/17 genes annotated to cAMP-mediated signalling (none enriched), all descendents of signalling.

^g^Includes 27/79 genes annotated to regulation of interphase, a descendent of regulation of the mitotic cell cycle (*p* = 1.23 × 10^−9^).

^h^Includes 18/67 genes annotated to regulation of mitosis (*p* = 4.88 × 10^−11^) and 6/27 genes annotated to attachment of spindle microtubules to kinetochore (*p* = 0.0351), descendents of regulation of the mitotic cell cycle.

^i^Includes 13/115 genes annotated to microtubule cytoskeleton, a descendent of cytoskeleton organization (*p* = 0.00706).

^j^Includes 4/9 genes annotated to Cdc42 signal transduction, a descendent of signalling (*p* = 0.006).

^k^Includes 7/52 genes annotated to actin cytoskeleton organization, a descendent of cytoskeleton organization (*p* = 8.09 × 10^−5^).

^l^Includes 13/115 genes annotated to microtubule cytoskeleton organization, a descendent of cytoskeleton organization (*p* = 2.12 × 10^−8^) and 5/5 genes annotated to gamma tubulin complex localization, a descendent of microtubule cytoskeleton organization (*p* = 3.11 × 10^−8^).

^m^Includes 3/11 genes annotated to carbon catabolite repression of transcription, a descendent of signalling (*p* = 0.00484).

^n^All 19 genes involved in mitochondrial tRNA metabolism.

**Table 3.** GO cellular components and complexes for all phenotype categories. Summary of the GO analysis for cellular components enriched within particular phenotype categories. For details see §5.5.2 and electronic supplementary material 1, tables S7*a–n* and S14. For further details, see table 2 legend. Footnotes are denoted by a–u. Red colour denotes enriched *p* ≤ 0.001; orange denotes moderately enriched *p* ≤ 0.01; light orange denotes weakly enriched *p* ≤ 0.1; light blue denotes no enrichment *p* ≤ 1; blank denotes number of genes is 0.

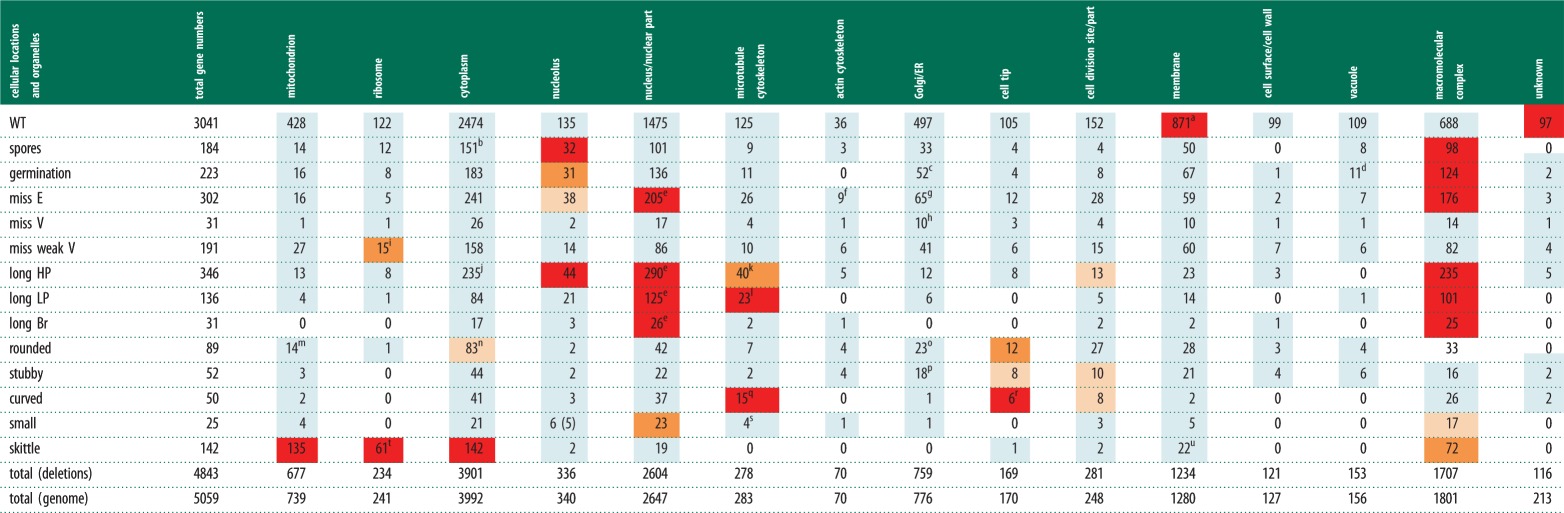

^a^Includes164/193 genes annotated to plasma membrane (*p* = 1.74 × 10^−9^) and 661/914 genes annotated to intrinsic to membrane (*p* = 5.45 × 10^−9^), both descendents of membrane.

^b^Includes 7/8 subunits of chaperonin containing T-complex (*p* = 1. 29 × 10^−7^) and 4/5 subunits of eukaryotic translation initiation factor 2B complex (*p* = 0.001).

^c^Includes 5/8 subunits of COP I coated vesicle membrane (*p* = 0.001).

^d^Includes 8/14 subunits of vacuolar proton-transporting V-type ATPase complex (*p* = 7.26 × 10^−6^).

^e^See table 4 for breakdown of nuclear complexes.

^f^Includes 5/8 subunits of Arp2/3 protein complex (*p* = 0.00922).

^g^Includes 5/7 subunits of oligosaccharyltransferase complex (*p* = 0.003), 3/3 subunits of glycosylphosphatidylinositol-*N*-acetylglucosaminyltransferase (GPI-GnT) (*p* = 0.05068), 6/11 subunits of TRAPP complex (*p* = 0.00417) and 4/4 subunits GARP complex (*p* = 0.00313).

^h^Includes 2/4 subunits of AP-1 adaptor complex (*p* = 0.02077).

^i^Includes 12/81 genes annotated to cytosolic large ribosomal subunit (*p* = 0.00936), a descendent of ribosome.

^j^Includes 5/6 subunits of elongator holoenzyme complex (*p* = 0.002) and 5/5 subunits of RNA cap binding complex (*p* = 0.0004).

^k^Includes 32/211 genes annotated to spindle pole body (*p* = 0.006), a descendent of microtubule cytoskeleton.

^l^Includes 20/211 genes annotated to spindle pole body (*p* = 0.00014), a descendent of microtubule cytoskeleton.

^m^Includes 2/4 subunits of mitochondrial sorting and assembly machinery complex (*p* = 0.22496).

^n^Includes 2/2 subunits of eRF1 methyltransferase complex (*p* = 0.03840).

^o^Includes 4/8 subunits of mannosyltransferase complex (*p* = 0.00081).

^p^Includes 8/194 genes annotated to ER membrane (*p* = 0.15), a descendent of Golgi/ER.

^q^Includes 4/10 genes annotated to equatorial MTOC (*p* = 0.00015) and 8/51 genes annotated to spindle pole body (*p* = 0.1), descendents of microtubule cytoskeleton.

^r^Includes 2/2 subunits of tea1 cell end complex (*p* = 0.008).

^s^Includes 4/211 genes annotated to spindle pole body (*p* = 1), a descendent of microtubule cytoskeleton.

^t^All 61 genes encode subunits of mitochondrial ribosome (61/70 subunits *p* = 3.98 × 10^−88^).

^u^Includes 20/173 genes annotated to mitochondrial membrane (*p* = 6.38 × 10^−6^), a descendent of membrane.

**Table 4.** A summary of the GO analysis for nuclear complexes enriched within the 3 *long* phenotype categories. For details, see §5.5.2 and electronic supplementary material 1, table S14. For further details, see table 2 legend. Footnotes are denoted by a–d. Red colour denotes enriched *p ≤* 0.001; orange denotes moderately enriched *p ≤*0.01; light orange denotes weakly enriched *p ≤*0.1; light blue is no enrichment *p ≤* 1; blank denotes number of genes is 0.

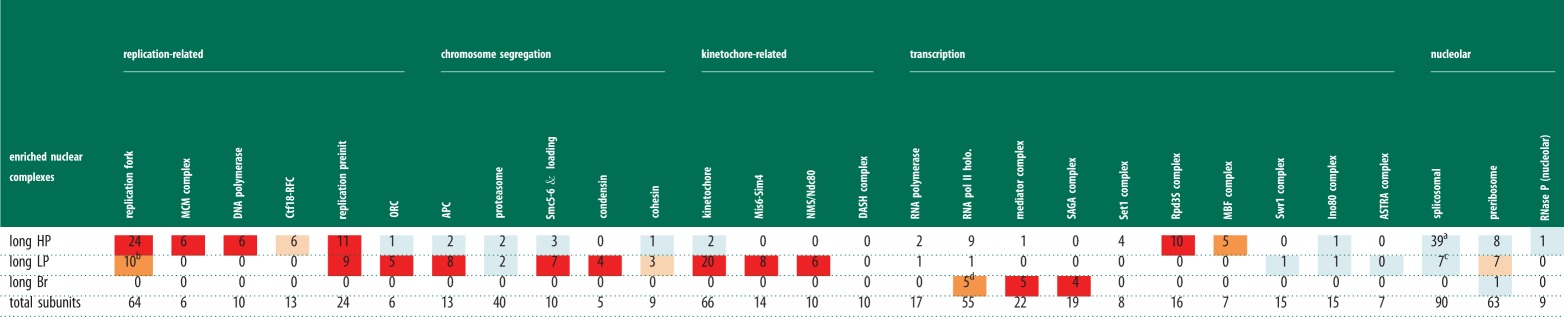

^a^Includes 13/26 subunits of U4/U6 × U5 tri-snRNP complex (*p* = 9.18 × 10^−7^), 6/7subunits of U6 SnRNP (*p* = 0.00017) and 6/6 subunits U2 snRNP (*p* = 0.0747).

^b^Includes 4/4 subunits of GINS complex (*p* = 7.14 × 10^−5^).

^c^Includes 4/15 subunits of U1 snRNP (*p* = 0.07660), 3/6 subunits of t-UTP complex (*p* = 0.04886) and 6/7 subunits of U6 SnRNP (*p* = 0.00017).

^d^All five genes encode subunits of holo TFIIH complex (5/10 subunits *p* = 1.08 × 10^−7^).

To identify genes required for the cell cycle and cell shape, we focused on the 11 cell shape categories. These phenotypes and their relevance for the cell cycle and cell shape are described in the following sections. To demonstrate the use of this resource, we describe a more detailed cytological analysis of a subset of mutants altered in cell shape. In addition, we screened the library of haploid viable deletion mutants for hydroxyurea (HU) sensitivity and identified new genes implicated in the DNA checkpoint preventing entry into mitosis when DNA replication is incomplete or DNA is damaged.

### 3.2. Gene deletion mutants with an elongated cell phenotype

A long cell phenotype identifies cells blocked in progression through interphase of the cell cycle or cytokinesis (see §2). Gene deletion mutants with this phenotype were assigned to three categories, long high penetrance (*long HP*; 346/4843), long low penetrance (*long LP*; 136/4843) and long branched (*long Br*; 31/4843) (figure 1 and table 1 and electronic supplementary material 1, tables S4 and S5*g–i*). These three categories totalled 513 genes required for cell cycle progression, and we conclude that 10 per cent of fission yeast genes are required directly or indirectly for progression through interphase or cytokinesis (513/4843). Four hundred and sixty-seven genes had a strong elongated deletion phenotype and a further 46 genes were included that were only weakly elongated when deleted (see §5.1). Of the 513 genes in the *long* categories, 66.5 per cent (341 genes) were essential for viability compared with 26.2 per cent for all genes, and 85.57 per cent (439 genes) were conserved in human. This indicates that many of the genes identified in this study are likely to be important for understanding the cell cycle in other more complex eukaryotes.

All three *long* categories were enriched for nuclear localization (table 3). The *long Br* set was enriched for cytokinesis and transcription and included genes encoding subunits of the RNA polymerase II holoenzyme, mediator and SAGA complexes (tables 2 and 4). As expected the *long HP* and *long LP* sets were both overrepresented for genes involved in DNA metabolism and the regulation of mitotic cell cycle (table 2). The *long HP* set was also enriched for processes and complexes involved in mRNA metabolism (particularly splicing), RNA biogenesis, transcription and DNA replication initiation, and 6/6 genes encoding subunits of the MCM complex (see table 4 and legend for details). By contrast, the *long LP* set was enriched for chromosome segregation and for genes encoding subunits of kinetochore complexes, including Mis6-SIM4 (8/14 subunits), Ndc-Mis-Spc (6/10 subunits), condensin (4/5 subunits) and APC (8/13 subunits) (tables 2 and 4). These differences in enrichment indicate that *long HP* is a good classifier for genes involved in progress through interphase, whereas *long LP* is more specific for genes associated with progress through mitosis (table 2 footnotes g,h). Mutants that block in mitosis usually display an irregular less defined cell shape but are not elongated, whereas an elongated phenotype is characteristic of an interphase block, so one possibility is that the *long LP* gene set is enriched for a subset of mitotic genes that are also required for interphase progression, with some cells arresting in mitosis and other cells in interphase. Together, these three *long* phenotype categories (*long HP*, *long LP* and *long Br*) define gene sets important for progression through interphase or cytokinesis. The *long LP* gene set is in addition enriched for a set of mitotic genes, which may also be required during interphase.

### 3.3. Cell cycle mutants without a long phenotype

Not all genes previously known to be required during interphase have an elongated phenotype when deleted. We found that DNA replication genes *cdc23, ssb1, pol1, cdc18, cdt1, rad4* and four genes encoding core subunits of the RFC (*rfc2, rfc3, rfc4* and *rfc5*) were all annotated to the misshapen essential set (*miss E*; table 1 and electronic supplementary material 1, table S5*d*). Some of these genes are known to be required for both DNA replication and the DNA checkpoint, and mutant strains enter mitosis with incompletely replicated DNA [16–20]. The *miss E* category also contains genes required for mitosis, for example, the kinetochore protein Nnf1 and pericentrin Pcp1. In total, 57 genes were annotated to one or more cell cycle process (table 2 and electronic supplementary material 1, table S6*d*). We conclude that the *miss E* phenotypic class will be a good starting point to screen for further genes required for both DNA replication and the DNA checkpoint as well as for genes required during mitosis.

Another group of previously known cell cycle genes exhibit a wee phenotype, with viable cells dividing at a shorter length and a smaller cell volume than WT cells. A partial gene deletion library of viable haploid mutants has already been screened, and 18 wee mutants identified [21]. In this study, we identified by visual examination 25 genes with a small cell deletion phenotype; this *small* set consisted of 11 non-essential genes and 14 essential genes (see the electronic supplementary material 1, table S5*m*). The 11 non-essential genes included nine of the 18 previously identified wee genes and were mainly those with a stronger wee deletion phenotype [21]. The two additional genes encode a predicted 26S proteasome non-ATPase regulatory subunit (SPCC18.17c), which has been found (F. Navarro and P. Nurse 2012, personal communication) to be shorter but also wider and so divided at a WT cell volume, and a mitochondrial inheritance GTPase (*dml1*), which is potentially a new wee gene. The 14 essential genes were enriched for tRNA metabolism (see table 2 for summary and the electronic supplementary material 1, tables S6*m* and S7*m* for details), including genes encoding subunits of the RNase P and mitochondrial RNase P, which have roles in RNA processing [22], tRNA 2’-*O*-ribose methyltransferase, tRNA-specific adenosine deaminase (2/2 subunits) and the tRNA-specific splicing endonuclease (2/4 subunits). It is possible that these non-viable small mutants, like other similar small size viable mutants identified in budding yeast that affect growth [23], may only indirectly affect cell cycle progression.

### 3.4. New cell cycle genes

Our genome-wide screen has identified 513 genes with a long cell deletion phenotype and thus required for the cell cycle in fission yeast. Previously, 158 fission yeast genes that generate elongated cells when deleted have been reported and annotated in PomBase (http://www.pombase.org/) [24]. To validate this qualitative visual approach to identify new cell cycle genes, we compared these 158 genes (see the electronic supplementary material 1, table S8*a*) with the 513 fission yeast cell cycle genes from this screen (see the electronic supplementary material 1, table S5*g–i*) and found that 147 of the 158 genes were also identified in our screen (see figure 2*b* and electronic supplementary material 1, table S9). The 366 genes not previously reported as elongated when deleted included 90 genes with an existing cell cycle GO annotation and 276 genes with no previously known cell cycle role (see the electronic supplementary material 1, tables S8*b* and S8*c*). The majority of these 276 genes (230/276) are annotated to GO processes which have previously been linked to the cell cycle, suggesting that the 276 genes are true positives and identify new cell cycle genes. The 230 new genes involved in cell-cycle-related processes included genes required for ribosome biogenesis, splicing and nucleotide metabolism (table 5). For example, seven genes (*dfr1, adk1, hpt1, dea2, dut1, dcd1, tmp1*) are concerned with various nucleotide metabolism pathways. Two previously identified cell cycle genes, budding yeast CDC8 (*tmp1* orthologue) [25], and the fission yeast *cdc22* (ribonucleotide reductase) [26] are also required for nucleotide metabolism. Given the cell cycle role of these two genes, the other genes identified here may also be important for maintaining the nucleotide levels needed for cell cycle progression.

**Table 5.** GO process annotations for 276 novel cell cycle genes. These 276 genes were not previously known to be involved in the cell cycle in fission yeast. Two hundred and thirty genes are annotated to other GO processes that have accepted links to the cell cycle in fission yeast and 29 genes had a GO process annotation not related to the cell cycle. Only 17 genes were of completely unknown function.

process in fission yeast	no. genes
GO processes cell-cycle-related	230
nucleocytoplasmic transport	13
mRNA metabolic process and splicing	73
ribosome biogenesis and cytoplasmic translation	37
Transcription	78
DNA repair, recombination, telomere maintenance	14
vesicle-mediated transport	5
modification by small molecule conjugation	3
nucleotide metabolism	7
GO processes not cell-cycle-related	29
small molecule metabolic pathways	13
miscellaneous	16
unknown process	17
fission-yeast-specific	4
fungal-specific	4
conserved to humans	9

The remaining 46 genes include 17 genes unstudied in any organism (nine of which are conserved in humans), and 29 genes that have existing GO annotations to processes or pathways not previously linked to the cell cycle. Of these, 13 genes are involved in a number of different metabolic pathways, including amino acid, carbohydrate and phospholipid metabolism. We investigated whether any of these genes had genetic or physical interactions with genes implicated in the cell cycle using the BioGRID Interaction database [27]. Several genes showed such interactions (see the electronic supplementary material 1, table S10). For example, a predicted pyruvate decarboxylase SPAC1F8.07c interacts with a wee gene *zfs1* [21,28]. It is possible that these 13 metabolic cell cycle genes may act as regulatory links between small molecule biosynthesis pathways and the cell cycle.

### 3.5. Comparison with a human cell cycle gene set

To examine the overlap between cell cycle genes in fission yeast and human, we identified a set of 521 human genes proposed to be involved in the cell cycle [12], and which have a fission yeast orthologue (see the electronic supplementary material 1, table S11*a* and §5.5.3). The 521 fission yeast orthologues of these human genes were compared with 614 fission yeast genes with an existing mitotic cell cycle annotation, including all genes so far annotated to the mitotic cell cycle either by inference or experiment (see the electronic supplementary material 1, table S11*b* and §5.5.1; http://www.pombase.org/). There were 113 genes common to both gene sets (see figure 2*c* and electronic supplementary material 1, table S11*c*). We also compared the 521 gene set with the 276 new cell cycle genes from this study and identified a further 43 genes in common (see figure 2*c* and electronic supplementary material 1, table S11*d*). Therefore, in total, 156 of the 521 conserved genes were involved in the cell cycle in both organisms (29.9%), a similar level to other inter-species comparisons, human/worm at 36 per cent and human/fly at 38 per cent [12,14,29,30]. Possible reasons why these inter-species comparisons in a variety of studies show such a low overlap are considered in §4.

### 3.6. Cell cycle checkpoint genes

**Figure 3.**
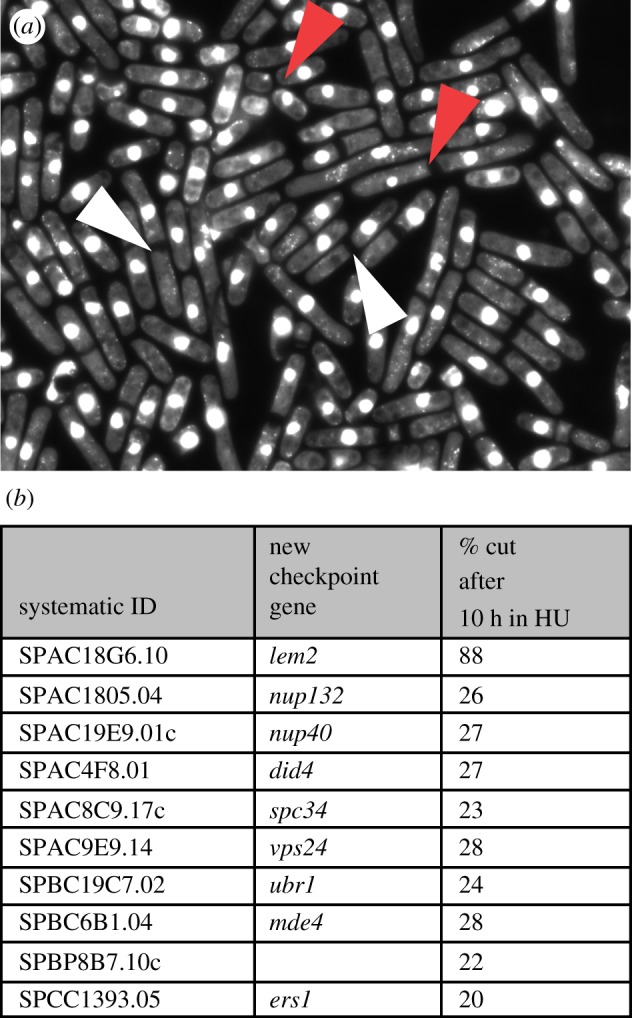
New HU-sensitive cut genes. (*a*) *lem2* deletion mutant cells stained with DAPI after growing for 8–10 h in the presence of HU. Examples of anucleated cells can be seen (white arrow) and cells with unequally segregated chromatin (red arrow). (*b*) New checkpoint genes identified in this study.

To identify new DNA checkpoint genes, we screened 2983 viable gene deletion mutants for those that failed to block the cell cycle in the presence of the ribonucleotide reductase inhibitor HU [31–33]. We further screened these HU-sensitive mutants for those with a cut phenotype [34], where cells fail to block cell cycle progress and enter mitosis generating chromosome segregation defects. We identified 132 mutants sensitive to at least 5 mM HU (see §5.2 and electronic supplementary material 1, table S12). In the presence of HU, deletion mutants of eight genes had greater than 60 per cent cells with a cut phenotype (see the electronic supplementary material 1, table S12, column O). Of these, deletion mutants of *hus1, rad1, rad3, rad26, rad17* and *rad9*, which have been previously been shown to be required for establishment of the DNA checkpoint, did not elongate prior to entering mitosis [33]. Deletion mutants of the remaining two genes (*ddb1, lem2*) initially became elongated but eventually entered mitosis and displayed a cut phenotype (figure 3*a*). Ddb1 is necessary for stabilizing DNA replication forks and is involved in regulating the replication checkpoint kinase Cds1 [35], and a *lem2* mutant has previously been shown to be sensitive to HU [36]. Mutants of a further 24 genes showed a lower level of cut cells (between 20 and 60%) after 10 h in HU (see the electronic supplementary material 1, table S12, column P). Of these, nine genes (*nup132, nup40, did4, spc34, vps24, ubr1, mde4*, *utp16* and *ers1*) are newly identified as HU-sensitive genes with a cut phenotype.

In this study, we have identified one new S phase checkpoint gene, *lem2,* which has a strong cut deletion phenotype, and nine genes with a lower penetrance cut deletion phenotype, which may influence maintenance of the S phase checkpoint (figure 3*b*).

### 3.7. Cell shape mutants

Cell shape mutants other than those with a long cell phenotype have been used to identify genes important for generating the normal rod-shape of a fission yeast cell. Previous studies in fission yeast have identified orb mutants that are spherical because cells fail to grow in a polarized manner, ban (banana) mutants that have a curved or bent cell phenotype because cells no longer orientate the growth zones at 180° along the long axis of the cell, and tea (tip elongation aberrant) mutants, which form a new growth zone in the wrong place often at 90° to the long axis of the cell [2,3]. Other cell shape mutants included bottle- or skittle-shaped cells and more generally misshapen cells [37]. We screened the deletion mutants for shape defects using the following seven categories to describe the mutant phenotypes (see figure 1 and electronic supplementary material 1, table S5*d–f*,*j–l*,*n* for gene lists). (i) *rounded*, which includes the typical orb mutants and also mutants that are more rounded than WT but not completely spherical. (ii) *stubby*, which look shorter and wider than WT but are mainly rod-shaped. These two categories showed a degree of overlap, with some mutants showing both phenotypes. (iii) *curved*, which includes the ban mutants and the tea mutants. During vegetative growth, tea mutants have only a low level of T-shaped cells with most cells having a curved phenotype. (iv) *skittle:* one end of cell the is wider than the other end. A total of 333 genes (6.9% of total genes) showed these specific alterations in cell shape when deleted and are thus important for the generation of normal cell shape. A further less well-defined group called *misshapen* showed an ill-defined potato-like shape or a mixture of other shapes. These fell into three further subgroups: (v) viable misshapen mutants (*miss V*), (vi) viable misshapen mutants, which have a weak phenotype (*miss weak V*) and (vii) essential misshapen mutants (*miss E*). There were 524 genes with these more general misshapen deletion phenotypes. In total, 857/4843 genes (17.7%) showed altered cell shape when deleted (table 1) and 668 of these are conserved in humans (77.9%). No additional cell shape phenotypes were identified compared with earlier work, suggesting that there may only be a restricted number of shapes that a fission yeast cell can adopt.

The *rounded, stubby* and *curved* sets are all enriched for genes implicated in cell polarity and for localization at the cell tip (tables 2 and 3), *rounded* and *stubby* sets for cell wall organization, and the *stubby* set for cytokinesis and actin cytoskeleton organization (table 2, footnote k). All 14 genes in the *curved* set annotated to the cytoskeleton organization category are involved in microtubule cytoskeleton organization (see table 2, footnote l) and are also enriched at the cell tip (table 3). Therefore, we predict that unknown genes with a curved deletion phenotype when deleted are likely to be involved in microtubule-related processes. Similarly, the stubby phenotype is likely to be associated with genes that affect actin processes. Genes which generate a skittle phenotype when deleted were enriched for mitochondrial organization; nearly, 50 per cent (118/241) of the total genes annotated to mitochondrion organization were in the *skittle* category. We found that 19 genes were required for mitochondrial tRNA metabolism (table 2) and 61 genes for the mitochondrial ribosome, suggesting that mitochondrial translation underlies the skittle phenotype. The *miss E* category was enriched for genes required for lipid metabolism (35 genes), 15 of which are involved in glycosylphosphatidylinositol (GPI) anchor biosynthesis (table 2, footnote a; electronic supplementary material 1, table S6*d*). GPI anchor proteins affect cell wall integrity, and loss of these proteins can result in a misshapen cell phenotype [38]. In higher eukaryotes, GPI anchor proteins have been implicated in the sorting of membrane proteins important for cell polarization [39], and so, in fission yeast, GPI anchor proteins could also be implicated in cell polarization.

**Figure 4.**
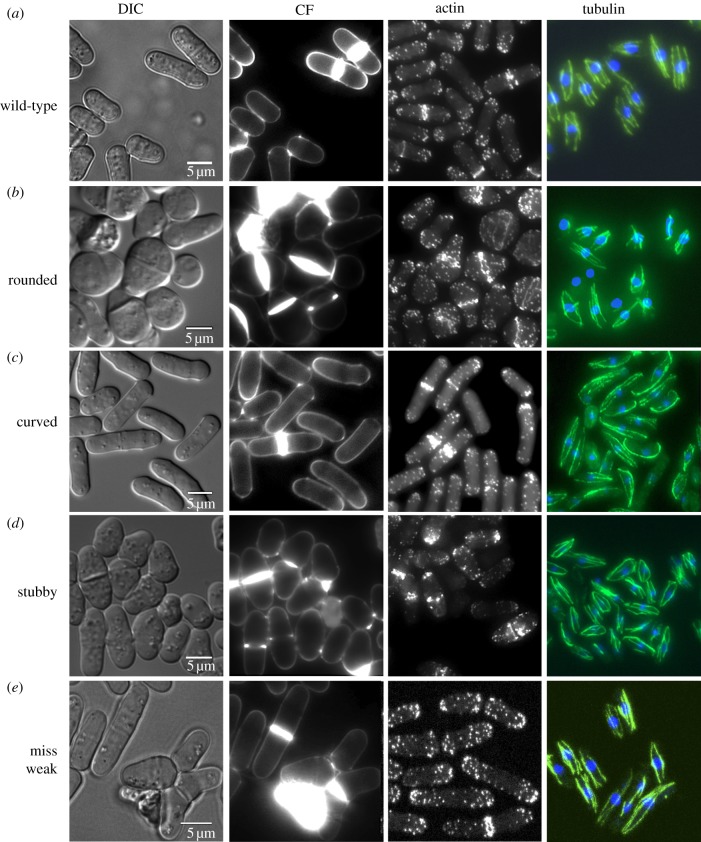
Cytological analysis of novel cell shape mutants. Examples of viable mutants from four cell shape phenotype categories analysed for defects in the cytoskeleton, growth pattern or cell wall. (*a*) Wild-type. (*b*) *meu29Δ.* (*c*) *yaf9Δ.* (*d*) *spc2Δ.* (*e*) *tlg2Δ*. DIC, differential interference contrast; CF, calcofluor used to stain the cell wall and septum. For details, see the electronic supplementary material 2 and electronic supplementary material 1, table S13.

To investigate whether gene deletions that cause cell shape changes also have defects in the cytoskeleton, bipolar growth pattern or the cell wall, we analysed 54 previously uncharacterized viable cell shape mutants. These were a subset of 352 non-essential genes in the *miss V*, *miss weak V, rounded, stubby, curved* and *skittle* categories (table 1). We found that a total of 35 strains had defects in the cytoskeleton and/or the cell wall, bipolar growth or cell separation defects and included 26 mutants with actin or microtubule defects (see figure 4 and electronic supplementary material 1, table S13 and electronic supplementary material 2 for details). These results indicate that further screening of viable deletion mutants, even those with a weak phenotype, will be a useful approach to identify and characterize genes involved in the cytoskeleton.

## 4. Discussion

We have visually screened 4843 gene deletion mutants in fission yeast, 95.7 per cent of all protein coding genes, and have identified near genome-wide sets of the genes required for the cell cycle and cell shape, the first systematic description for a eukaryote. The long cell phenotype in fission yeast defines cells blocked in cell cycle progression during interphase or cytokinesis and so is an effective way to identify genes required for these stages of the cell cycle. GO enrichment analysis has shown that the *long HP* and *long Br* categories were enriched for genes previously identified as being required during interphase or cytokinesis respectively. The *long LP* set included genes previously known to be required during mitosis and we suggest that these genes may represent a subgroup of mitotic genes that have additional roles during interphase. Several genes required for the cell cycle were found in the *miss E* gene set suggesting that further analysis of this set may also identify new genes required during interphase or mitosis.

We identified 513 cell cycle genes in total, 276 of which were not previously known to have a role in the cell cycle. Of these new genes, 230 were annotated to GO processes previously implicated in the cell cycle, thus identifying new links between the cell cycle and these processes. These included genes required for nucleocytoplasmic transport, mRNA metabolic process (specifically splicing), ribosome biogenesis and nucleotide metabolism (table 5). There were 46 new cell cycle genes not annotated to a process previously associated with the cell cycle, 13 of which are involved in small molecule metabolism. Frequently, only one or two genes were identified for a specific metabolic pathway. For example *ect1* is predicted to encode ethanolamine-phosphate cytidylyltransferase, which is rate-limiting for synthesis of CDP-ethanolamine, an important step in phospholipid biosynthesis [40]. We speculate that these genes may encode proteins linking different aspects of metabolism, such as metabolite levels or flux, to the cell cycle.

Studies using RNAi in metazoan organisms have identified sets of genes required for cell cycle progression. However, these overlap only to a limited extent between various intra- and inter-species comparisons in a range from 10 to 38 per cent [12]. Our analysis comparing cell cycle genes in fission yeast and human identified 156/521 orthologous gene pairs (29.9%) involved in the cell cycle in both organisms, a similar percentage overlap to that found in other inter-species studies. A possible reason for the rather limited overlap of cell cycle genes in a wide range of studies may be because gene knockdowns using RNAi can result in varying levels of gene product and thus more variation in cellular phenotype. Our analysis is based on gene deletions that generally eliminate the entire gene function, thus reducing phenotypic variability. Inter-species comparisons may also be limited because different phenotypes may be used in different organisms to infer a specific cell cycle defect, and these are not always directly comparable. A cell cycle defect during interphase in fission yeast produces an easily identifiable highly consistent long cell phenotype, which can be used to reliably identify interphase cell cycle genes. Many of the cell cycle genes identified in fission yeast have a conserved cell cycle role in other eukaryotes and, so the *long* cell cycle gene set identified in this study is likely to be useful to uncover additional cell cycle genes conserved across species.

To catalogue genes required to generate and maintain the correct cell shape, we identified categories of deletion mutants with specific cell shape defects. GO analysis showed that genes within these groups could be used to identify genes implicated in the actin cytoskeleton (*stubby*), the microtubule cytoskeleton (*curved*) and mitochondrial function (*skittle*). The *skittle* category suggests that there is an uncharacterized mechanism influencing cell shape, which is affected by defects in mitochondrial organization and translation. The distribution of mitochondria in a cell is dependent on microtubules [41], and binding of mitochondria to microtubules can moderate microtubule dynamics [42], raising the possibility that defective mitochondria may affect microtubules thus leading to cell shape changes. In humans, a number of diseases including deafness and muscle pathologies are linked to defects in mitochondrial protein synthesis [43]. The link we have identified in this study between mitochondria and cell shape suggests that cell shape changes could be an underlying cause of some of these pathologies in humans. We also showed that some genes from the *miss weak V* set, although only exhibiting a mild shape change when deleted, have cytoskeletal defects. This suggests that genes with this deletion phenotype will be a good source of new genes affecting the actin and microtubule cytoskeleton.

A limited range of defined cell shapes were identified in the genome-wide gene deletion screen. It appears that only a restricted range of cell shapes is possible for the fission yeast cell, perhaps reflecting topological constraints in cellular organization, related to the cell wall or the cytoskeleton, for example. Future comparisons with other organisms that have been screened for cell shape defects [10,11,13] will help identify the genes and processes required to generate or maintain eukaryotic cell shape.

Our work provides the first near genome-wide sets of gene deletions that influence the eukaryotic cell cycle and cell shape. This qualitative classification of genes according to cell shape phenotypes, based on deletion mutants, provides a resource that will be a good starting point for further studies in fission yeast and for the identification of equivalent gene functions in other eukaryotic organisms.

## 5. Material and methods

### 5.1. Phenotype analysis of the genome-wide set of gene deletions

The 4843 deletion strain collection used for this analysis consists of 4825 strains described by Kim *et al.* [7] plus 18 additional gene deletion mutants as shown in the electronic supplementary material 1, table S2. Changes in gene dispensability from Kim *et al.* [7] for nine reconstructed strains and 12 re-analysed strains are shown in the electronic supplementary materia 1, table S3. All growth conditions and media were used as described by Moreno *et al*. [44], unless otherwise stated. Spores were generated as described for gene dispensability analysis [7]. All strains were coded, and a blind analysis was conducted. Between two and four isolates for each heterozygous diploid deletion mutant were independently sporulated. A visual examination of the phenotypes of both deletion G418-resistant spores and WT G418-sensitive spores following free spore analysis was carried out after 1 and 2 days following plating on non-selective YES plates at 25°C and 32°C. The presence of both G418-sensitive and -resistant spores allowed a comparison of the deletion mutant with WT. Any phenotypic differences from WT that could be detected by eye were described as the putative deletion phenotype. After 2 days, colonies were replica plated onto YES plates containing G418 (Sigma) at 25°C and 32°C to confirm the gene deletion phenotype by linkage to the G418-resistant phenotype.

The final deletion phenotype categories for a genome-wide set of genes were generated as follows, a GO analysis of the *long* group was compared with a GO analysis of the subdivisions *long HP, long LP* and *long Br*. These subdivisions formed biologically significant subgroups of the *long* group and so these three categories were used for further analysis. The same type of GO analysis was used for the *rounded* and *stubby* groups as these formed biologically distinct categories, although there is also overlap between the phenotypes of these two categories. The *misshapen* group was divided into essential and non-essential genes, because viable mutants may be more useful for identifying genes required for cell shape where as the misshapen phenotype observed in the essential group may be less specific for a cell shape defect given that the cells are dying or dead. The remaining categories *WT, spores, germination, skittle, curved* and *small* could not be usefully further subdivided by their phenotype.

To estimate the minimal cell length increase detectable, we measured the cell length of 34 viable gene deletion mutants described as long after visual screening on plates and which had not previously been implicated in the cell cycle (see the electronic supplementary material 1, table S1 column J). We could detect cells at least 10 per cent or longer compared with WT (approx. 15.6 µm) and, so a 10 per cent or greater increase in cell length was used as the criteria for a long phenotype. The cut-off between high penetrance and low penetrance was 30 per cent long cells. This was estimated using inviable mutants that formed microcolonies showing a mixture of long and WT/short cells. For these mutants, 30 per cent  or less of the cells had a long phenotype.

To validate this approach, we compared the 513 genes from the three *long* categories with 158 cell cycle genes reported in PomBase as long. We identified 147/158 (93%) of these genes, suggesting that the remainder of the genes in our *long* category are also likely to be involved in the cell cycle. Furthermore, only 46/513 were not annotated to a GO category previously linked to the cell cycle.

Cells showing different cell shape defects were photographed using a Zeiss Axioskop microscope with a CF plan X50/0.55 objective and a Panasonic DMC-LX2 camera. Spores from representative strains were plated on to YES solid medium and allowed to germinate or form small colonies before being photographed.

### 5.2. Screen for new DNA checkpoint genes

The growth of 2983 viable deletion mutants from Bioneer version 1 were screened on YES agar plates for 24–48 h either in the presence of 2.75 or 5.5 mM HU or without HU and scored on a scale of strong (+++), medium (++), weak (+) or no sensitivity, depending on their ability to grow on different HU concentrations compared with no HU (see the electronic supplementary material 1, table S12). To check whether the 132 HU-sensitive mutants were also involved in the DNA checkpoint preventing mitosis, cells were grown in liquid cultures with 11 mM HU and screened for a cut phenotype using 4’,6-diamidino-2-phenylindole (DAPI) to visualize the nucleus.

### 5.3. Cell length measurements

Cells were grown to mid-exponential growth (2 × 10^6^ to 1 × 10^7^ cells ml^−1^) in YES liquid medium at 32°C (or 25°C where appropriate) and photographed using a Zeiss Axioplan microscope with 100× objective and a COHU CCD camera. Cell lengths of 30 septated cells were measured using ImageJ.

### 5.4. Phenotypic analysis and cytoskeleton analysis of viable shape mutants

For the initial characterization, cells were grown at 18°C, 25.5°C, 29°C and 34°C on minimal and YES medium plates, and cell morphology analysed by differential interference contrast (DIC) microscopy. For further characterization, strains showing morphological defects were grown in liquid rich medium at 25°C to mid-log phase or in the conditions at which each strain showed the strongest phenotype by DIC microscopy. Septa and pattern of cell growth was visualized with 35 µg ml^−1^ calcofluor staining (fluorescent brightener; Sigma). For WT cells, the septation index was 15 per cent (*n* = 500 cells) and 30.1 per cent of cells showed monopolar growth (*n* = 300 cells). Nuclei were visualized with 0.2 µg ml^−1^ DAPI (Sigma) or 100 µm ml^−1^ IP (Sigma) staining. Actin staining was as described by Pelham & Chang [45] using AlexaFluor 488-phalloidin (Molecular Probes). For anti-tubulin immunofluorescence, cells were fixed in methanol at −80°C and further processed as described by Hagan & Hyams [46]. Primary antibodies were anti-tubulin ((TAT-1; 1 : 80 dilution) followed by Alexa 488 goat anti-mouse secondary antibody (Molecular Probes). Microscopy was performed at 23–25°C, either with an Axioplan 2 microscope (Carl Zeiss, Inc.) equipped with a CoolsnapHQ camera (Roper Scientific) or with a Leica TCS SL confocal microscope. Data were acquired using the 100× objective taking seven *z*-sections with 0.5 μm spacing.

### 5.5. Bioinformatics analysis

#### 5.5.1. Identification of genes already implicated in mitotic cell cycle processes

To identify genes involved in the mitotic cell cycle in fission yeast, we used fission yeast GO data from 26 September 2011 (http://www.pombase.org/), and selected the set of protein coding genes annotated to:

GO:0000278 mitotic cell cycle,

GO:0000910 cytokinesis,

GO:0006261 DNA-dependent DNA replication, and

GO:0000075 cell cycle checkpoint.

Minor adjustments were made to this dataset to remove three known false positives (SPAC343.17c, SPBC19F8.02, SPAC5D6.08c) and add three known false negatives (SPAC23H4.11c, SPAC26A3.03c SPAC23H4.18c). The complete list is provided in the electronic supplementary material 1, table S11*b*.

#### 5.5.2. Gene Ontology enrichment analysis

GO enrichments were performed using GO term finder (http://go.princeton.edu/cgi-bin/GOTermFinder with ontology and annotations from 26 September 2011). Threshold *p*-values of 0.001, 0.01 and 0.1 were used to identify specific enrichments. Bonferroni correction was used.

GO slim categories presented in tables 2, 3 and 4 refer to the GO IDs in the electronic supplementary material 1, table S14. All GO terms and *p*-values for each phenotype set are provided in the electronic supplementary material 1, tables S6 and S7. Genes where number of annotations = 1 were obtained using GO term Mapper (http://go.princeton.edu/cgi-bin/GOTermMapper).

The background set was the 4843 gene set used this study.

#### 5.5.3. Comparison with human cell cycle genes

A list of 1351 human cell cycle genes was extracted from a study by Kittler *et al.* [12] and mapped to current Ensembl IDs (79 of the human identifiers had been retired and were no longer linked to extant genes). The remainder were mapped to 521 fission yeast orthologues using Ensembl Compara 10/11/2011 (http://genome.cshlp.org/content/19/2/327.long).

## 6. Acknowledgements

This work was supported by Cancer Research UK, the Wellcome Trust, Breast Cancer Research Foundation, KRIBB, NRF grants (nos. 2012M3A9D1054666 and 2011-0016688) from the Korea Ministry of Science, ICT & Future Planning (MSIP). We are very grateful to Midori Harris, Mark McDowall, Kim Rutherford and Juan-Juan Li for providing help with the analysis. We are also indebted to all members of the Cell Cycle Laboratory, particularly Francisco Navarro, for reading of the manuscript and helpful suggestions. There are no conflicts of interest resulting from this work.

## Supplementary Material

Hayles et al Supplementary Material RSOB-13-0053

## Supplementary Material

Table S1

## Supplementary Material

Table S5

## Supplementary Material

Table S6

## Supplementary Material

Table S7

## Supplementary Material

Table S8

## Supplementary Material

Table S10

## Supplementary Material

Table S11

## Supplementary Material

Table S12

## Supplementary Material

Table S13

## Supplementary Material

Table S14
