# Supplementary material for: A genome-wide resource of cell cycle and cell shape genes of fission yeast
Source: Open Biol. 2013 May;3(5):130053. doi: 10.1098/rsob.130053 (PMC3866870; doi:10.1098/rsob.130053)
Supplement: Hayles et al Supplementary Material RSOB-13-0053 [file rsob130053-s1.docx]

**Supplementary Material 1**

**Table S1 General information for 4843 fission yeast deletion mutants screened in this study**

For general information see column headings. Classification of genes using the phenotype terms WT, Spores, Germination, Misshapen essential, Misshapen viable, Misshapen weak viable, Long high penetrance, Long low penetrance, Long branched, Rounded, Stubby, Curved, Small and Skittle, shown in Column H. For a description of each phenotype term see Table S4. Each gene was classified by a single phenotype category based on the most penetrant or strongest deletion phenotype (Column G). Cells were only described as wild type (WT) when no other phenotype was observed. A description of the deletion phenotype for each gene (column H), using controlled vocabulary, demonstrates the variation in the deletion phenotype observed for genes annotated to one particular phenotype category. Using the Excel filter function column G can also be used to select for genes with any of the phenotypes that were not analysed further see Table S4 **Terms for other phenotypes observed**. Cell length measurements for weak long genes are shown in Column J.

**Table S2 Dispensability of 18 genes not previously included in the set of genome-wide gene deletions**

| **Cosmid ID**  **Newly constructed deletions** | **Gene name** | **Published phenotype** | **Dispensability this study** | **% deleted** |
| --- | --- | --- | --- | --- |
| SPCC320.04c |  | No data | V | 99 |
| SPCC4B3.16 | tip41 | Viable | V | 83 |
| SPCPJ732.03 | meu15 | No data | V | 100 |
| SPAC22E12.16c | pik1 | No data | E | 94 |
| SPAC11E3.12 |  | No data | V | 100 |
| SPAC750.08c |  | No data | V | 100 |
| **Cosmid ID Genes deleted <40%** | **Gene name** | **Published phenotype** | **Dispensability this study** | **% deleted** |
| SPAC26A3.08 | smb1 | No data | E | 6 |
| SPAC3G9.03 | rpl231 | No data | V | 9 |
| SPAC824.06 | tim14 | No data | E | 5 |
| SPAC977.06 |  | No data | V | 8 |
| SPBC1347.01c | rev1 | No data | V | 2 |
| SPBC16E9.01c | php4 | No data | V | 4 |
| SPBC17A3.02 |  | No data | V | 34 |
| SPBC405.01 | ade1 | No data | V | 3 |
| SPCC1235.01 |  | No data | V | 4 |
| SPCC1682.16 | rpt4 | No data | V | 5 |
| SPCC24B10.06 |  | No data | V | 32 |
| SPAC9E9.04 |  | No data | V | 40 |

Genes shown here include 6 genes with newly constructed gene deletion mutants and 12 genes that were not analysed by Kim et al 2010 [[1](#_ENREF_1)] for gene dispensability as they were less than 40% deleted. **Table S3 Gene dispensability changes from Kim et al 2010**

| **Cosmid ID** | **Gene name** | **Kim et al 2010 dispensability deletion dataset** | **Kim et al 2010 dispensability analysis dataset** | **Dispensability**  **This study** | **Comments** |
| --- | --- | --- | --- | --- | --- |
| SPAC144.08 |  | V | V | E | Deletion remade |
| SPAC21E11.06 | tif224 | V | V | E | Deletion remade |
| SPAC23A1.16c |  | V | V | E | Re-analysed |
| SPAC3A11.08 | pcu4 | V | V | E | Deletion remade confirmed different to pub |
| SPAC3H5.11 |  | V | V | E | Deletion remade |
| SPAC6C3.09 |  | V | V | E | Deletion remade |
| SPAC6G10.05c |  | V | V | E | Deletion remade |
| SPBC337.13c | gtr1 | V | V | E | Re-analysed |
| SPBC577.10 |  | V | V | E | Deletion remade |
| SPCC1739.13 | ssa2 | V | V | E | Re-analysed |
| SPCC4B3.14 | cwf2 | V | V | E | Re-analysed |
| SPBC800.09 | sum2 | E | E | V | Re-analysed |
| SPCC1739.12 | ppe1 | E | E | V | Deletion remade - same as pub |
| SPBC16C6.09 | ogm4 | E | V | V | Re-analysed - same as pub |
| SPAC13D6.0 | alp11 | V | E | E | Re-analysed - same as pub |
| SPAC1565.06c | spg1 | V | E | E | Re-analysed -same as pub |
| SPBC32F12.06 | pch1 | V | E | E | Re-analysed -same as pub |
| SPCC1620.11 | nup97 | V | E | E | Re-analysed -same as pub |
| SPCC18.04 | pof6 | V | E | E | Re-analysed -same as pub |
| SPCC777.10c | ubc12 | V | E | E | Re-analysed -same as pub |
| SPCC1739.07 |  | V | E | V | Deletion remade confirmed different to pub |

3 gene deletion strains have been reconstructed as they had a different phenotype to previously published data. We find that 2 are still different to published data and one is now the same as previously published. Deletion mutants of 6 other genes that were non-essential (V) were remade and these genes are now essential (E). 12 gene deletion mutants were re-analysed and found to have a different dispensability, 7 of these now have the same phenotype as previously published deletion mutants.

Pub = previously published independently of the genome wide gene deletion project

**Table S4 Cell phenotype terms and description**

| **Cell Phenotype term** | **Cell Phenotype term description** | **FYPO identifier** |
| --- | --- | --- |
| **Terms used for analysis** |  |  |
| WT | Normal cell morphology  (wild type) | 0000672 |
| Spores | Normal spore morphology most fail to germinate | 0000306 |
| Germination | Normal germinated spore morphology most fail to divide | 0000310 |
| Misshapen E | Uniformly lumpy or a range of different shapes cells/spores are inviable | 0001511 |
| Misshapen V | Uniformly lumpy or a range of different shapes. Cells are viable | 0001510 |
| Misshapen weak V | Uniformly lumpy or a range of different shapes. Phenotype is weak, cells are viable | 0001510 |
| Long HP | Distance between cell ends is longer than normal in greater than 20% of cells | 0001122 |
| Long LP | Distance between cell ends is longer than normal in <20% of cells | 0001122 |
| Long Br | Distance between cell ends is longer than normal and cells branch next to septum at right angles to the long axis of cell | 0001512 |
| Rounded | Spherical or oval shaped cell | 0000946 |
| Stubby | Shorter and wider than normal | 0000024 |
| Curved | Cell ends not apposed at 180° along long cell axis | 0000016 |
| Small | Lower cell volume than normal | 0000645 |
| Skittle | Tapered at one end | 0000014 |
| **Terms for other phenotypes observed** |  |  |
| Lysis | Cell wall integrity compromised causing cytoplasm to be released | 0000647 |
| Septated | Increased number of septated cells compared to normal or  multiseptated or misplaced septum | 0000650  0000117 |
| Swollen | Increased in all dimensions compared to normal | 0000025 |
| T-shaped | Single branch at right angles to the long axis of cell | 0000013 |
| Vacuolated | Wild type cells with abnormal vacuolated appearance | 0001581 |

The cell phenotypes terms used to describe each gene deletion mutant with a brief description of the phenotype. A definition of each phenotype can be found using the FYPO ID at [www.berkeleybop.org/obo/**ontology**/**FYPO**](http://www.berkeleybop.org/obo/ontology/FYPO)**,** <http://www.pombase.org/>). In this study phenotype terms may describe the phenotype of spores, germinated spores or cells. A more detailed description of the phenotype of each deletion mutant is in Table S1 column G. Deletion mutants with a cell phenotype of interest can be selected for using Table S1 column H for the phenotype, column J for gene dispensability and column A for systematic ID of the deleted gene. As well as the 14 major cell phenotypes see **Terms used for analysis,** some deletion mutants had more than one phenotype eg. SPAC16C9.05 which is described as long, curved. These genes were assigned to the most penetrant deletion phenotype. Some deletion phenotypes that were observed were not studied further as independent phenotype categories (see **Terms for other phenotypes observed**). Any genes showing these additional phenotypes were also assigned to one of the 14 major phenotypes. For those interested in these phenotypes the genes can be selected for using Table S1 column G and selecting for the appropriate term shown in **Terms for other phenotypes observed**.

**Table S5 A-N** **Cell Phenotype category gene lists**

Gene lists for the 14 phenotype categories

A Wildtype

B Spores

C Germination

D Misshapen essential

E Misshapen viable

F Misshapen weak viable

G Long high penetrance

H Long low penetrance

I Long branched

J Rounded

K Stubby

L Curved

M Small

N Skittle

**Table S6 A-N GO processes for genes in each cell phenotype category**

For the GO analysis the following parameters were used

Aspect P

P-value cutoff 1

Calculate FDR Yes

Regulation links followed Yes

Bonferroni correction Yes

Uploaded gene list phenotype category_names_only_number of genes (see A-N below

Annotation file gene_association.GeneDB_Spombe

Uploaded background file back_ground_set_names_only_4843

Evidence codes used IEA (3515), IEP (422), IGI (1231), NAS (1631), IPI (1175), ND (2139), IC (1446), ISS (10811), RCA (722), IMP (2916), IDA (9671), TAS (1093)

A Wildtype

B Spores

C Germination

D Misshapen essential

E Misshapen viable

F Misshapen weak viable

G Long high penetrance

H Long low penetrance

I Long branched

J Rounded

K Stubby

L Curved

M Small

N Skittle

**Table S7A-N** **GO component for genes in each cell phenotype category**

For the GO analysis the following parameters were used

Aspect C

P-value cutoff 1

Calculate FDR Yes

Regulation links followed Yes

Bonferroni correction Yes

Uploaded gene list phenotype category_names_only_number of genes (see A –N below)

Annotation file gene_association.GeneDB_Spombe

Uploaded background file back_ground_set_names_only_4843

Evidence codes used IEA (3515), IEP (422), IGI (1231), NAS (1631), IPI (1175), ND (2139), IC (1446), ISS (10811), RCA (722), IMP (2916), IDA (9671), TAS (1093)

A Wildtype

B Spores

C Germination

D Misshapen essential

E Misshapen viable

F Misshapen weak viable

G Long high penetrance

H Long low penetrance

I Long branched

J Rounded

K Stubby

L Curved

M Small

N Skittle

**Table S8** **New cell cycle genes in fission yeast**

Gene lists for the comparison of 513 genes with a long cell deletion phenotype with 158 genes previously published as having a long cell deletion phenotype.

A 158 genes previously published as long when deleted

B 90 genes identified in this study which have a previous GO cell cycle annotation

C 276 new cell cycle genes from this study

**Table S9 Published cell cycle genes not identified in this study**

| **Systematic ID** | **Gene name** | **Function** | **Published phenotype** | **Phenotype this study** |
| --- | --- | --- | --- | --- |
| **GO cell cycle annotation** |  |  |  |  |
| SPAC1486.04c | alm1 | Medial ring protein Alm1 | Deletion. High penetrant long cells | Curved and is also described as Long weak |
| SPAC16A10.04 | rho4 | Rho family GTPase Rho4 | Deletion. Septation defect and long cells | WT only 50% deleted |
| SPBC1709.05 | sks2 | Heat shock protein, ribosome associated molecular chaperone Sks2 | Disruption  High penetrant long cells | WT |
| SPBC1734.14c | suc1 | Cyclin-dependent protein kinase regulatory subunit Suc1 | Disruption. Low penetrance long cells | Miss E |
| SPBC1A4.05 | blt1 | Regulation of the G2/M transition Blt1 | Deletion High penetrant long cells | WT only 76% deleted |
| SPBC31F10.13c | hip1 | Hira protein, histone chaperone Hip1 | Deletion. High penetrant long cells | WT |
| SPBC646.13 | sds23 | Inducer of sexual development Sds23 Moc1 | Deletion. Long cells at high and low temperature | WT |
| SPBC660.13c | ssb1 | DNA replication factor A subunit Ssb1 | Deletion. Low penetrant long cells | Miss E |
| SPBC6B1.09c | nbs1 | Mre11 complex subunit | Deletion. High penetrant long cells | WT only 66% deleted |
| **No GO cell cycle annotation** |  |  |  |  |
| SPAC1006.03c | red1 | RNA elimination defective protein Red1 | Deletion. Low penetrant long cells | Miss V |
| SPAC1F3.07c | rsc58 | RSC complex subunit Rsc58 | Deletion. Low penetrant long cells | Miss weak V |

Eleven genes previously published as having a long cell phenotype when deleted were not identified in our 513 **Long** gene set. 9 genes also have a GO cell cycle annotation and 2 genes are previously published as having a long cell deletion phenotype but currently have no GO cell cycle annotation. Several of these mutants are gene disruptions rather than deletions, which may account for the different phenotypes. In others the long phenotype is low penetrance and may have been missed by us. For 3 genes *blt1, rho4* and *nbs1* it may be that our strains are incorrect as they are not complete gene deletions (see TableS1, column D. For the *hip1* gene the phenotype is different between our strain and the previously published strain.

**Table S10**  **New cell cycle genes involved in small molecule metabolism**

BioGrid analysis to identify genetic or physical interactions of new cell cycle genes not previously known to be involved in the cell cycle. For interacting genes we have shown the systematic ID and the gene name where known.

**Footnotes**

1. Rate limiting step in synthesis of CDP-ethanolamine.

2. Catalytic subunit of serine palmitoyltransferase (SPT), which catalyzes the commitment step in the synthesis of sphingolipids.

3. Converts sphingosine-1-phosphate to sphingosine. Sphingosine-1-phosphate is a cofactor of the human E3 ubiquitin ligase TRAF1 [[2](#_ENREF_2)] and is important for pro survival processes.

4. *slm9* implicated in the mitotic control [[3](#_ENREF_3)].

5*. zfs1* suppressor of *cdc16* [[4](#_ENREF_4)] and identified as a wee gene in fission yeast [[5](#_ENREF_5)].

6. Both *gpd1* and *tdh1* encode glyceraldehyde-3-phosphate dehydrogenase and act in different metabolic pathways but both interact with the stress response pathway.

7. Cyk3, transglutaminase-like superfamily, required for cytokinesis in fission yeast.

8. Plays a central role as a glucosyl donor in cellular metabolic pathways.

**Table S11 Comparison with human cell cycle gene orthologues**

Gene lists from the comparison of 521 orthologues implicated in the cell cycle in humans, 614 genes with a GO cell cycle annotation in fission yeast and 276 new cell cycle genes from this study.

A. Human cell cycle gene orthologues -521 genes

B. Genes with GO cell cycle annotation in fission yeast -614 genes

C. Genes identified as cell cycle genes in humans and fission yeast -113 genes

D. New cell cycle genes from this study identified as cell cycle genes in humans -43 genes

**Table S12** **HU sensitivity screen**

Analysis of 132 genes identified in a screen of 2983 gene deletion mutants for HU sensitivity and cut phenotype. 132 HU sensitive genes were identified (Column C) by sensitivity to different HU concentrations, strong (+++), medium (++), weak (+). These genes were then tested for a cut phenotype in the presence of 11mM HU Columns D-K. Column L, previously reported HU sensitivity in budding yeast, Column M, previously reported HU sensitivity in fission yeast. Further information about each gene shown to have a cut phenotype when deleted is shown in Columns N-P. Column N genes previously reported as cut**,** Column O genes showing greater than 60% CUT, Column P showing 20-60% CUT.

**Table S13 A summary of the analysis of 54 gene deletion mutants with a shape defect**

Summary of the analysis of a set of morphology mutants previously uncharacterized for actin cytoskeleton, microtubule cytoskeleton, cell wall, cell growth pattern and septation. Mutants are grouped according to their deletion phenotype on plates.

1. Previously published gene without cytoskeleton characterization
2. Gene not previously published, new morphology gene
3. weak morphology defect with no evidence of actin, microtubule, growth pattern or cytokinesis/cell wall defects

MT = microtubules, ts = temperature sensitive, pen = penetrance. YES = Yeast extract agar adenine, leucine, histidine and uracil

For details see Supplementary Material 2 **Analysis of a set of viable deletion mutants with a cell shape defect.**

+ actin cytoskeleton, microtubule cytoskeleton, cell wall, cell growth pattern or septation defect

- no actin cytoskeleton, microtubule cytoskeleton, cell wall, cell growth pattern or septation defect

**Table S14 Mappings for GO processes**

Mappings for biological processes, locations and complexes used in Table 2, Table 3A and Table 3B. * significant enrichments reported for either term.

**Supplementary Material 2**

**Analysis of a set of viable deletion mutants with a cell shape defect**

We analysed 54 viable morphology mutants and 35/54 (64.8%) strains showed some type of defect (cytoskeleton defects, cytokinesis defects or monopolar pattern of growth). Immunofluorescence analysis of the cytoskeleton in these morphological mutants shows that 26/35 (74.3%) strains had actin or microtubule cytoskeleton defects. The remaining 9/35 (25.7%) strains, show either monopolar pattern of growth as judged by calcofluor staining, cell wall defects (cell lysis) or defects in cell-cell separation (accumulation of septated cells/ septation index greater than WT).

Among the genes whose deletions show cytoskeleton defects, cytokinesis defects or monopolar pattern of growth, 15/35 (42.8%) have not been previously studied and a further 20/35 (57.2%) strains, have been previously studied but the morphological defects have not been characterized. Mutants have been categorized according to their phenotype on plates.

Details can be found our website at

<http://www.london-research-institute.org.uk/research/paul-nurse-jacqueline-hayles/resources/paper-supplementary>

**References**

1. Kim, D.U., et al., *Analysis of a genome-wide set of gene deletions in the fission yeast Schizosaccharomyces pombe.* Nat Biotechnol, 2010. **28**(6): p. 617-23.

2. Alvarez, S.E., et al., *Sphingosine-1-phosphate is a missing cofactor for the E3 ubiquitin ligase TRAF2.* Nature, 2010. **465**(7301): p. 1084-8.

3. Kanoh, J. and P. Russell, *Slm9, a novel nuclear protein involved in mitotic control in fission yeast.* Genetics, 2000. **155**(2): p. 623-31.

4. Beltraminelli, N., M. Murone, and V. Simanis, *The S. pombe zfs1 gene is required to prevent septation if mitotic progression is inhibited.* J. Cell Sci., 1999. **112 Pt 18**: p. 3103-14.

5. Navarro, F.J. and P. Nurse, *A systematic screen reveals new elements acting at the G2/M cell cycle control.* Genome Biol, 2012. **13**(5): p. R36.

6. Dodgson, J., et al., *Functional genomics of adhesion, invasion, and mycelial formation in Schizosaccharomyces pombe.* Eukaryotic Cell, 2009. **8**(8): p. 1298-306.

7. Guerin, M. and A.J. Parodi, *The UDP-glucose:glycoprotein glucosyltransferase is organized in at least two tightly bound domains from yeast to mammals.* J. Biol Chem., 2003. **278**(23): p. 20540-6.

8. Dawson, K., et al., *Loss of regulators of vacuolar ATPase function and ceramide synthesis results in multidrug sensitivity in Schizosaccharomyces pombe.* Eukaryotic Cell, 2008. **7**(6): p. 926-37.

9. Spahr, H., et al., *Analysis of Schizosaccharomyces pombe mediator reveals a set of essential subunits conserved between yeast and metazoan cells.* PNAS U S A, 2001. **98**(21): p. 11985-90.

10. Buchanan, L., et al., *The Schizosaccharomyces pombe JmjC-protein, Msc1, prevents H2A.Z localization in centromeric and subtelomeric chromatin domains.* PLoS genetics, 2009. **5**(11): p. e1000726.

11. Hogan, C.J., et al., *Fission yeast Iec1-ino80-mediated nucleosome eviction regulates nucleotide and phosphate metabolism.* MCB, 2010. **30**(3): p. 657-74.

12. Nicolas, E., et al., *Distinct roles of HDAC complexes in promoter silencing, antisense suppression and DNA damage protection.* Nature Structural & Molecular Biology, 2007. **14**(5): p. 372-80.

13. Bayne, E.H., et al., *Stc1: a critical link between RNAi and chromatin modification required for heterochromatin integrity.* Cell, 2010. **140**(5): p. 666-77.

14. Gao, Q., et al., *A non-ring-like form of the Dam1 complex modulates microtubule dynamics in fission yeast.* Proceedings of the National Academy of Sciences of the United States of America, 2010. **107**(30): p. 13330-5.

15. Lejeune, E., et al., *The chromatin-remodeling factor FACT contributes to centromeric heterochromatin independently of RNAi.* Current Biology 2007. **17**(14): p. 1219-24.

16. Roguev, A., et al., *High conservation of the Set1/Rad6 axis of histone 3 lysine 4 methylation in budding and fission yeasts.* J. Biol.Chem., 2003. **278**(10): p. 8487-93.

17. Chen, J.Q., et al., *The fission yeast inhibitor of growth (ING) protein Png1p functions in response to DNA damage.* J. Biol. Chem., 2010. **285**(21): p. 15786-93
